# An Overview of Preventive Strategies and the Role of Various Organizations in Combating Antimicrobial Resistance

**DOI:** 10.7759/cureus.44666

**Published:** 2023-09-04

**Authors:** Yogendra P Shelke, Nandkishor J Bankar, Gulshan R Bandre, Dattu V Hawale, Pratibha Dawande

**Affiliations:** 1 Microbiology, Bhaktshreshtha Kamalakarpant Laxmanrao Walawalkar Rural Medical College, Sawarda, IND; 2 Microbiology, Jawaharlal Nehru Medical College, Datta Meghe Institute of Higher Education and Research, Wardha, IND; 3 Biochemistry, Datta Meghe Medical College, Datta Meghe Institute of Higher Education and Research, Nagpur, IND; 4 Pathology, Datta Meghe Medical College, Datta Meghe Institute of Higher Education and Research, Nagpur, IND

**Keywords:** infection control, antimicrobial stewardship, global health, antibiotics, antimicrobial resistance

## Abstract

The rise of antimicrobial resistance (AMR) is a major global public health threat due to excessive and inappropriate use of antibiotics and is responsible for prolonged illness, longer hospital stays, and economic burden to society. This article aims to review the factors, role of antimicrobial stewardship, preventive strategies, and role of various organizations in combating AMR. Three major factors of AMR are inappropriate and excessive utilization of antibiotics, nonadherence to infection control measures, and the emergence of pathogens that are resistant to multiple drugs. Antimicrobial stewardship initiatives play a vital role in promoting judicious and targeted utilization of antimicrobials, thereby safeguarding their efficacy and mitigating the emergence of resistance. Implementing such programs optimizes patient outcomes by ensuring that individuals receive the most suitable therapeutic interventions. International organizations have a vital role to play in addressing AMR by promoting the responsible use of antimicrobials, developing new drugs, and improving surveillance systems. As AMR's impact grows, it is critical to take a collaborative and interdisciplinary approach to mitigate its consequences effectively.

## Introduction and background

Antimicrobial resistance (AMR) is one of the top global public health issues, which is primarily caused by the misuse and overuse of antimicrobials. AMR is responsible for prolonged illness, longer hospital stays, and an economic burden to society. AMR is making antibiotics more and more ineffective, necessitating the urgent need for new antibacterials [1-3].

AMR refers to the capacity of bacteria to proliferate and disseminate within the human body, even when exposed to medications intended to eradicate or impede their growth [4]. The rise and dissemination of AMR pose a substantial public health concern because of their potential to impede effective infection treatments, prolong illness duration, necessitate extended hospital stays, and escalate healthcare expenditures [1]. In addition, it has the potential to contribute to morbidity and mortality rates, specifically among susceptible cohorts encompassing young children, elderly individuals, and individuals who are immunocompromised. AMR has significant implications for both individuals and communities, manifesting as an augmented burden of contagious illnesses, diminished efficacy of medical interventions reliant on antimicrobial prophylaxis, and a notable economic toll characterized by diminished productivity and escalated healthcare expenses [5]. In developing countries, misuse of antimicrobials is promoted through non-regulated supply chains and the sale of over-the-counter medications without prescription [6]. Some people deliberately stop their antimicrobials when their signs and symptoms begin to subside or due to side effects, and may come to the hospital with recurrent infections by more virulent and resistant strains [7]. This article aims to review the factors, role of antimicrobial stewardship, preventive strategies, and role of various organizations in combating AMR.

## Review

Causes of AMR

Three major factors of AMR are inappropriate and excessive utilization of antibiotics, nonadherence to infection control measures, and the emergence of pathogens that are resistant to multiple drugs. The widespread and indiscriminate use of antibiotics, spanning human and animal populations, imposes selective pressures that facilitate the development of bacteria with resistance capabilities.

Misuse and Overuse of Antibiotics

The improper and excessive utilization of antibiotics represents a significant challenge in the realm of public health. In instances where antibiotics are used without genuine necessity or in an inappropriate manner, they can foster the propagation of AMR strains. Subsequently, these resistant strains can disseminate to other individuals, posing further risks [8,9]. The aforementioned circumstances can present challenges in the effective management of infections, resulting in prolonged periods of illness, extended hospitalizations, and a subsequent increase in healthcare expenditures [9,10]. To mitigate the emergence and spread of AMR, it is imperative to exercise the judicious use of antibiotics, reserving their application solely for cases where they are genuinely warranted. Additionally, adherence to the prescribed dosage and treatment duration is essential to ensure the most effective and efficient therapeutic outcomes. Moreover, adhering to proper hygiene protocols and actively pursuing vaccination can substantially minimize reliance on antibiotics, thereby further curbing the necessity for their usage [4,11].

Failure of Infection Control Practices

Infection control is a set of measures to prevent communicable diseases [12]. Maintaining stringent infection control practices is of paramount significance, especially within healthcare environments, given the heightened susceptibility of patients to infections due to their underlying health conditions. Neglecting proper infection control protocols can result in the dissemination of communicable diseases and entail grave ramifications, including outbreaks of diseases, as well as detrimental effects on the reputation of healthcare establishments [13-15]. To avert the breakdown of infection control measures, adherence to established protocols and guidelines, maintenance of hygienic and disinfectant settings, and observance of exemplary hygiene practices are crucial. This entails regular hand washing, employing appropriate measures, and proficiently managing and disposing of infectious materials. Additionally, it is imperative to guarantee that all healthcare personnel receive comprehensive training in infection control practices and are held responsible for their meticulous implementation [16,17].

Emergence of Multiple-Drug-Resistant (MDR) Pathogens

MDR microorganisms have the capacity to withstand numerous categories of antimicrobial agents, thereby impeding effective treatment approaches [2,18,19]. Prevalence of MDR microorganisms can give rise to an extended period of ailment and prolonged hospitalization, alongside a surge in expenses related to healthcare. The emergence of superbugs can occur as a consequence of the inept or excessive utilization of antimicrobial agents, thereby facilitating the proliferation of antibiotic-resistant bacterial strains through selective pressures [8,20]. To mitigate the probability of MDR, it is imperative to employ antibiotics solely when their necessity is unequivocal and to administer them at the correct dosage and duration of therapy [9]. Implementing adequate hygiene practices and employing efficacious infection control measures are important in averting the dissemination of antibiotic-resistant microorganisms [19,21].

Global trends in AMR

The distribution of AMR exhibits geographical and regional variation, highlighting an inequitable burden across the globe. According to the World Health Organization (WHO), AMR affects approximately 75% of the population globally [22]; however, its impact is unevenly distributed, primarily affecting specific regions and demographics. Vulnerable groups include individuals residing in low- and middle-income countries, individuals living with HIV, individuals afflicted by chronic diseases, and individuals receiving medical attention within healthcare facilities [23]. Moreover, the WHO has provided an estimation indicating that a staggering number of over 700,000 fatalities occur globally on an annual basis due to infections characterized by antibiotic resistance. Alarming predictions suggest that this figure is anticipated to surge exponentially, reaching 10 million deaths in a year by 2050 [23-27].

AMR poses a considerable challenge in effectively combating numerous infectious diseases, rendering their treatment increasingly arduous [3]. Certain significant global phenomena in AMR encompass the rise of antibiotic-resistant bacteria, exemplified by methicillin-resistant *Staphylococcus aureus* (MRSA) and carbapenem-resistant *Enterobacteriaceae* (CRE). The dissemination of multidrug-resistant tuberculosis (MDR-TB) represents another prevailing occurrence. AMR instills mounting apprehension within the context of sexually transmitted infections, notably gonorrhea. Additionally, it progressively compromises the efficacy of novel antimicrobial agents, including antivirals and antifungals [18,28]. It is imperative for countries to collaborate on a global scale to combat this predicament effectively, as AMR transcends geographical boundaries and exhibits the potential for seamless cross-border propagation [29].

Mechanisms of AMR

AMR emerges when microorganisms acquire the capacity to endure exposure to antimicrobial agents that are specifically formulated to eradicate them or impede their proliferation [30]. Common mechanisms by which microorganisms develop resistance to antimicrobial drugs include genetic mutation, genetic transfer, alteration of drug target, drug inactivation, and efflux pumps, and biofilm formation.

Genetic Mutation

Microbial entities possess the ability to acquire resistance against antimicrobial agents through genetic alterations, referred to as mutations, within their DNA sequences. These mutations can transpire spontaneously or may be provoked by the presence of antimicrobial substances [31].

Genetic Transfer

Microorganisms possess the capability to develop AMR via genetic transfer which facilitates the acquisition of resistance traits. This phenomenon can occur through diverse mechanisms, including horizontal gene transfer, which involves the direct transfer of genetic material between organisms. Additionally, resistance genes can be acquired through plasmids or transposons, which serve as carriers capable of conveying resistance genes from one organism to another [32,33].

Alteration of Drug Target, Drug Inactivation, and Efflux Pumps

Some microorganisms can acquire resistance through alteration of the drug target, which denotes the precise location within the organism that the drug is intended to combat, thus rendering it less vulnerable to the effects of the drug attack [32,34,35]. Bacteria can produce enzymes that exhibit the capacity to neutralize antimicrobial agents, thereby leading to a loss of efficacy [32,36]. Certain microorganisms possess efflux pumps, a class of proteins that can expel antimicrobial agents from within the cell. This mechanism impedes the accumulation of these therapeutic substances to the effective concentration necessary to eradicate the organism [37-40].

Biofilm Formation

Certain microbial entities have the capacity to create biofilms, characterized by slender strata comprising microorganisms adhered to a surface and encompassed by an extracellular matrix. These biofilms possess the ability to shield against antimicrobial agents, resulting in heightened challenges in eradicating associated microorganisms [41-43].

Role of antimicrobial stewardship

Antimicrobial stewardship encompasses the conscientious utilization of antimicrobial agents, including antibiotics, to mitigate the occurrence and dissemination of AMR [44,45]. The process encompasses the optimization of therapeutic interventions for infectious ailments with a simultaneous reduction in the deleterious effects stemming from the administration of antimicrobial agents, specifically aimed at mitigating resistance development and adverse reactions [46,47].

Antimicrobial stewardship can enhance patient outcomes by curbing the occurrence and dissemination of AMR and preserving the efficacy of currently available antimicrobial agents [48,49]. These initiatives typically encompass the formulation of protocols governing the prudent utilization of antimicrobials, execution of measures to enhance the appropriateness of antimicrobial prescriptions, and surveillance of patterns pertaining to antimicrobial consumption and AMR [50].

Antimicrobial stewardship is of significant importance because of the adverse effects of the excessive and improper utilization of antimicrobials, which can lead to the emergence and proliferation of AMR. Antimicrobial stewardship initiatives play a vital role in promoting judicious and targeted utilization of antimicrobials, thereby safeguarding their efficacy and mitigating the emergence of resistance. By implementing such programs, patient outcomes can be optimized by ensuring that individuals receive the most suitable therapeutic interventions. Thus, the proper management of antimicrobials and balance between effective treatment and minimizing the development of resistance can be achieved [51-53]. Thus, antimicrobial stewardship guarantees the proper and efficient utilization of antimicrobial agents while simultaneously mitigating the likelihood of AMR development.

The key components involved in antimicrobial stewardship are education, surveillance, intervention, and evaluation (Table 1).

**Table 1:** Key components involved in antimicrobial stewardship

Sr No	Key Components	Description
1	Education	This is a crucial aspect of promoting responsible antimicrobial use, encompassing the dissemination of knowledge to healthcare professionals regarding the appropriate utilization of antimicrobials. Additionally, educating patients about the significance of adherence to prescribed antimicrobial regimens is imperative [54].
2	Surveillance	This encompasses the diligent observation of antimicrobial utilization, encompassing the prescription patterns of various medications and the corresponding medical indications for their administration [55].
3	Intervention	Strategies aimed at enhancing the utilization of antimicrobials may alter the drug regimen or modify the dosage and duration of treatment [56].
4	Evaluation	Periodic assessment is imperative to consistently appraise the efficiency of initiatives focused on antimicrobial stewardship and, if required, implement modifications accordingly [46].

Strategies for preventing AMR

To tackle AMR, it is important to adopt an interdisciplinary approach that acknowledges the intricate and interrelated nature of the contributing factors. Numerous strategies can be implemented to prevent AMR (Table 2).

**Table 2:** Strategies for preventing antimicrobial resistance AMR: antimicrobial resistance

Sr No	Strategies	Description
1	Prescribing antimicrobials only when they are necessary and appropriate	Overuse and inappropriate use of antimicrobials is one of the main drivers of AMR [9]. Healthcare professionals should only prescribe antimicrobials when they are necessary to treat a confirmed or suspected bacterial infection and should choose the most appropriate antimicrobial based on the susceptibility of the infecting microorganism [46,57].
2	Implementing antimicrobial stewardship	These programs may include educating healthcare professionals about appropriate antimicrobial use, implementing guidelines and protocols, and monitoring and tracking antimicrobial use and resistance patterns [53,58,59].
3	Isolating infected or colonized patients	This may involve placing the patient in a private room or using infection control measures such as hand hygiene and personal protective equipment [60,61].
4	Promoting vaccination	Vaccines can help to reduce the burden of infectious diseases and the need for antimicrobials. This is particularly important vaccine preventable diseases, such as *Streptococcus pneumonia*e and *Haemophilus influenzae* [62-64]
5	Developing new antimicrobials	Drug discovery is an important strategy for combating AMR. However, it is important to use these drugs responsibly to preserve their effectiveness [65,66].

Social and economic impact of AMR

AMR can have a massive social and economic impact. In terms of economics, AMR can lead to increased healthcare costs due to the need for more expensive or prolonged treatments, as well as the cost of managing outbreaks of resistant infections. AMR can also affect the agriculture industry, as antimicrobials are used to prevent and treat infections in animals [67]. Also, loss of productivity, negative impact on global trade, difficulty in controlling outbreaks, and negative impact on global health are some of the social and economic impact of AMR (Table 3).

**Table 3:** Social and economic impact of antimicrobial resistance AMR: antimicrobial resistance

Sr No	Potential Consequences	Description
1	Increased healthcare costs	AMR treatments can be more expensive and may require longer hospital stays or costly medical interventions [68-71].
2	Loss of productivity	AMR infections can lead to absenteeism from work or school, which can result in lost productivity and income [72].
3	Negative impact on global trade	AMR can affect the trade of agricultural products, such as meat, dairy, and produce, which can have economic consequences for farmers and the food industry [73,74].
4	Difficulty in controlling outbreaks	AMR infections lead to larger outbreaks, with serious consequences for public health [3].
5	Negative impact on global health	In developing countries, where access to healthcare and antimicrobials may be limited, the impact of AMR can be particularly severe [6,74].

Role of international organizations in addressing AMR

International organizations are uniquely positioned to address AMR, given that it is a global health threat that requires a collaborative and cross-sectoral approach. Several international organizations are working to address AMR.

WHO

The WHO is the United Nations agency responsible for international public health. It has developed a Global Action Plan that outlines several strategies for addressing AMR, including improving surveillance, promoting the development of new antimicrobials, and improving access to clean water and sanitation [75].

Food and Agriculture Organization of the United Nations (FAO)

The FAO works to improve food security and nutrition, and has recognized the importance of addressing AMR in the livestock sector. It has developed guidelines for the responsible use of antimicrobials in animal agriculture and is working to improve surveillance and data collection on antimicrobial use in this sector [76,77].

World Organization for Animal Health (WOAH)

The WOAH (founded as Office International des Epizooties (OIE)) is an international organization that promotes the health and welfare of animals and works to prevent and control animal diseases. It has developed guidelines for the responsible use of antimicrobials in animal health and is working to improve surveillance and data collection on antimicrobial use in this sector [78,79].

The World Bank

The World Bank is a financial institution that provides loans and grants to countries to support economic development and poverty reduction. It has recognized the economic consequences of AMR and has funded projects related to AMR, including efforts to improve surveillance and data collection and to promote the development of new antimicrobials [26,80].

Group of 20 (G20)

G20 has recognized the importance of addressing AMR and has committed to taking action to address this issue [81,82].

Global Antimicrobial Resistance Partnership (GARP)

GARP is a multi-sectoral partnership that brings together experts from a range of fields to address AMR [83,84].

International Council of Nurses (ICN)

The ICN is a professional organization that represents nurses globally. It works to address AMR through the promotion of antimicrobial stewardship and the development of guidelines and recommendations for the appropriate use of antimicrobials in healthcare settings [85,86].

Global Antimicrobial Resistance Surveillance System (GLASS)

GLASS is an initiative of the WHO that aims to strengthen global surveillance of AMR and improve the data available on the problem [87,88].

The roadmap for addressing AMR

"A Roadmap to Tackle the Challenge of Antimicrobial Resistance - A Joint Meeting of Medical Societies in India" was convened as a symposium during the second annual conference of the Clinical Infectious Disease Society in Chennai, India, in 2012. This groundbreaking assembly marked the inaugural congregation of medical societies in India, united with the objective of devising a comprehensive strategy to combat the global challenge of AMR from an Indian standpoint. Distinguished representatives from numerous medical societies across India, eminent policymakers from both central and state governments, delegates from the WHO, National Accreditation Board of Hospitals, Medical Council of India, Drug Controller General of India, and Indian Council of Medical Research, alongside prominent personalities within the Indian medical domain, were in attendance, collectively steering the discourse towards an effective response against AMR [89].

The WHO's Expert Committee on Selection and Use of Essential Medicines created the AWaRe classification system for antibiotics in 2017. It's an important tool for advancing antibiotic stewardship efforts worldwide. The classification system divides antibiotics into three categories: Access, Watch, and Reserve. This categorization takes into account the impact of various antibiotics and antibiotic classes on the development of AMR. It underscores the critical importance of using antibiotics judiciously and responsibly. The AWaRe classification is updated every two years. Its primary purpose is to monitor antibiotic consumption, establish benchmarks, and assess the outcomes of stewardship policies. The WHO 13th General Programme of Work from 2019 to 2023 outlines a country-level target. The target states that at least 60% of total antibiotic consumption should be attributed to antibiotics in the Access group. This effort aims to enhance the accessibility and appropriate use of antibiotics on a global scale [90].

Overall, international organizations play a vital role in coordinating efforts to address AMR and in providing support for countries to implement strategies to reduce the emergence and spread of resistance.

## Conclusions

AMR is a growing health threat. It requires an interdisciplinary approach and collaborative efforts to recognize and combat AMR, which is a serious health and economic burden that affects all sectors. International organizations and countries need to support the development and implementation of policies to reduce the inappropriate use of antibiotics in both humans and animals. Greater investment in research and development for new antibiotics is needed.
